# *Miscanthus* Grass as a Novel Functional Fiber Source in Extruded Feline Diets

**DOI:** 10.3389/fvets.2021.668288

**Published:** 2021-06-04

**Authors:** Shannon E. Finet, Bruce R. Southey, Sandra L. Rodriguez-Zas, Fei He, Maria R. C. de Godoy

**Affiliations:** Department of Animal Sciences, University of Illinois, Urbana, IL, United States

**Keywords:** cats, dietary fiber, fecal microbiota, *Miscanthus* grass, nutrient digestibility, post-biotics

## Abstract

Although dietary fiber is not considered an essential nutrient in a complete and balanced diet for felines, it provides a substrate for fermentation by gut microbiota, thus promoting gastrointestinal health through the production of fermentative metabolites, as well as improving laxation. The aim of this research was to evaluate the novel fiber source, *Miscanthus* grass (*Miscanthus giganteus*), in comparison with traditional fiber sources and their effects on fecal quality, apparent total tract digestibility (ATTD), fecal fermentative end products, and microbiota of healthy adult cats. Four dietary treatments were evaluated, differing in dietary fiber source. The diets were formulated to meet or exceed the AAFCO (2018) nutritional profile for adult cats and contained either cellulose (CO), *Miscanthus* grass fiber (MF), a blend of *Miscanthus* fiber and tomato pomace (MF + TP), or beet pulp (BP). The study was conducted using a completely randomized design with 28 neutered adult, domesticated shorthair cats (19 females and 9 males, mean age 2.2 ± 0.03 years; mean body weight 4.6 ± 0.7 kg, mean body condition score 5.6 ± 0.6). The experimental period comprised 21 days, and a fresh fecal and a total fecal collection were performed during the last 4 days of the trial period. Daily food intake (DM basis) was similar across all groups (*P* > 0.05). Additionally, treatment did not affect fecal output (as-is or DM basis), fecal score, or fecal pH (*P* > 0.05). Cats fed BP had significantly higher total dietary fiber ATTD than all the other treatments (*P* < 0.05) and the highest concentrations of total short-chain fatty acid, acetate, and propionate (*P* < 0.05), while butyrate concentrations were similar for all treatments (*P* > 0.05). Inclusion of dietary fibers was effective in modulating gut microbiota. Cats fed diets containing *Miscanthus* grass had greater α-diversity than cats fed BP. As no adverse effects on health, fecal quality, or ATTD of macronutrients were observed with the inclusion of 9% *Miscanthus* grass fiber or fiber blend, the data suggest that *Miscanthus* grass fiber and fiber blends are viable alternatives to the traditional dietary fiber sources used in commercial extruded feline diets, being most comparable to cellulose.

## Introduction

Since the 1950s, when the term “dietary fiber” was first introduced, several attempts have been made by the scientific community and regulatory bodies to provide a clearer and more encompassing definition of this term (1). This is because a diverse group of substances and ingredients fall under this umbrella term, which differ in many aspects including origin, physicochemical properties, and physiological effects. In 2016, the Food and Drug Administration issued a final ruling on the definition of “dietary fiber.” They defined it as “non-digestible soluble and insoluble carbohydrates (with 3 or more monomeric units), and lignin that are intrinsic and intact in plants; isolated or synthetic non-digestible carbohydrates determined by the Food and Drug Administration to have physiological effects that are beneficial to human health” (2). Many studies have shown the broad range of health benefits associated with dietary fiber intake by humans, with a major focus on gut health (3). This interest has spread to companion animal nutrition as well.

*Miscanthus* grass (*Miscanthus giganteus*) is an ingredient that has the potential to act as a novel dietary fiber source in companion animal diets. This grass is largely composed of insoluble fibers, making it compositionally similar to cellulose, and a potential base for blends with more fermentable fiber sources to develop desirable fiber matrices. It also contains naturally occurring xylooligosaccharides that may provide a prebiotic-like effect; however, more research is required to determine this. As an ingredient, *Miscanthus* grass offers many positive marketing attributes such as “natural,” non-GMO, non-by-product, and potentially organic. Limited data are available on the use of *Miscanthus* grass in monogastric diets, especially in regard to its effects on gastrointestinal health. Therefore, the goal of this research was to compare the effects of *Miscanthus* grass fiber to traditional dietary fiber sources and their effects on gastrointestinal tolerance, apparent total tract macronutrient digestibility, and fecal fermentative end products in adult cats fed extruded diets.

The most traditionally used sources of dietary fiber in companion animal diets are cellulose and beet pulp (4). These ingredients, like most fiber sources, have distinct fiber profiles that allow them to provide different physiological effects. Fiber characteristics such as viscosity, solubility, and fermentability determine the functional effects of various dietary fiber sources. Cellulose is made up almost entirely of insoluble, non-viscous fiber. Purified sources of cellulose are highly uniform in composition, while other commercially available sources may be by-products of other industries resulting in more variable compositions. Beet pulp has a mixed composition, consisting of viscous, non-viscous, soluble, and insoluble fibers. The ratios of these fiber portions can be inconsistent, leading to more variability in this product (4). It was hypothesized that the diet containing *Miscanthus* grass would produce results similar to the diet containing cellulose, with the addition of tomato pomace to the *Miscanthus* fiber blend leading to results that are intermediate between cellulose and beet pulp diets.

## Materials and Methods

### Diets, Animals, and Experimental Design

Four diets were formulated to meet or exceed the AAFCO nutrient profile for adult cats (*n* = 7 cats/treatment) (5). They were formulated with similar ingredient composition, except for the dietary fiber sources being tested, and to have similar nutrient composition and a targeted total dietary fiber (TDF) content of 15%. To achieve this target, the diets were formulated to contain 7% cellulose (CO), 9% *Miscanthus* grass fiber (MF), 7% *Miscanthus* grass fiber plus 2% tomato pomace (MF + TP), or 11% beet pulp (BP) (Table 1).

**Table 1:** Ingredient composition of treatments containing traditional and novel fiber sources for adult felines.

**Ingredient, % as is**	**Treatment**^****1****^			
	**CO**	**MF**	**MF + TP**	**BP**
Poultry by-product meal	40.31	40.00	38.31	37.81
Brewers rice	32.00	30.00	32.00	30.00
Poultry fat	8.50	8.81	8.50	9.00
Yellow corn	5.00	5.00	5.00	5.00
Corn gluten meal 60%	5.00	5.00	5.00	5.00
AFB palatant	1.00	1.00	1.00	1.00
Salt	0.50	0.50	0.50	0.50
Choline chloride	0.13	0.13	0.13	0.13
Potassium chloride	0.10	0.10	0.10	0.10
BHT antioxidant	0.10	0.10	0.10	0.10
Mineral premix^2^	0.18	0.18	0.18	0.18
Vitamin premix^3^	0.18	0.18	0.18	0.18
Cellulose	**7.00**	0.00	0.00	0.00
*Miscanthus* grass	0.00	**9.00**	**7.00**	0.00
Beet pulp	0.00	0.00	0.00	**11.00**
Tomato pomace	0.00	0.00	**2.00**	0.00

1 *CO, cellulose; MF, M-fiber; MF + TP, M-fiber + tomato pomace; BP, beet pulp.*

2 *Provided per kg diet: 17.4 mg manganese (MnSO_4_), 284.3 mg iron (FeSO_4_), 17.2 mg copper (CuSO_4_), 2.2 mg cobalt (CoSO_4_), 166.3 mg zinc (ZnSO_4_), 7.5 mg iodine (KI), and 0.2 mg selenium (Na_2_SeO_3_).*

3 *Provided per kg diet: 11,000 IU vitamin A, 900 IU vitamin D_3_, 57.5 IU vitamin E, 0.6 mg vitamin K, 7.6 mg thiamin, 11.9 mg riboflavin, 18.5 mg pantothenic acid, 93.2 mg niacin, 6.6 mg pyridoxine, 12.4 mg biotin, 1,142.1 μg folic acid, and 164.9 μg vitamin B_12_. The bold values represent the formulated inclusion levels of the traditional and novel dietary fiber ingredients that were evaluated in this study*.

All animal procedures were approved by the University of Illinois Institutional Animal Care and Use Committee. Twenty-eight neutered, adult domesticated shorthair cats were used in a completely randomized design. At the start of the experiment, all cats were adapted to the CO diet for 7 days. After this control adaptation period, all cats were randomly assigned to one of the four treatment diets and were fed for 21 days to maintain body weight. The CO group consisted of two males and five females [age 2.2 ± 0.3; body weight (BW) 4.3 ± 0.4; body conditions score (BCS) 5.4 ± 0.2]; the BP group, two males and five females (age 2.2 ± 0.3; BW 4.4 ± 0.6; BCS 5.4 ± 0.6); the MF group, three males and four females (age 2.2 ± 0.2; BW 4.9 ± 0.6; BCS 5.9 ± 0.6); and the MF + TP group, two males and five females (age 2.2 ± 0.3; BW 4.7 ± 1.0; BCS 5.6 ± 0.9). During the last 4 days of this period, a total fecal and a fresh fecal collection were performed.

Cats were group housed for 20 h of the day and individually housed in stainless steel cages for 4 h per day for feeding. Feeding occurred twice a day from 08:00 to 10:00 and 15:00 to 17:00. Cats had free access to water at all times. Food refusals were weighed and recorded after each feeding. Body weights and body condition scores were measured and recorded weekly. Cats were housed in the Edward R. Madigan Laboratory in a climate-controlled room with a 14-h light and 10-h dark cycle. Human socialization periods took place at a minimum of two times per week, and cats had access to behavioral enrichments such as scratching posts.

### Sample Collection and Preparation

For the duration of the 4-day fecal collection period, cats were housed individually. All feces were collected during this time and composited by cat to determine total fecal output. Each sample also was evaluated for fecal score on a five-point scale (1 = hard, dry pellets; 2 = hard formed, remains firm and soft; 3 = soft, formed and moist stool; 4 = soft, unformed stool; or 5 = watery, liquid that can be poured), and then samples were stored at −20°C for later analysis to determine the apparent total tract digestibility (ATTD) of macronutrients.

A fresh fecal sample was collected from each cat within 15 min of defecation during the 4-day fecal collection period. These samples were also evaluated for fecal pH and score and dry matter. Then, they were aliquoted to determine ammonia, short-chain fatty acid (SCFA), branched-chain fatty acid (BCFA), phenol, and indole concentrations. To determine dry matter (DM) content, duplicates of ~2 g of feces were dried in a forced air oven at 105°C. For determination of fecal ammonia, SCFA, and BCFA concentrations, 3 g of each fresh sample was placed in a Nalgene bottle and mixed with 3 ml of 2 N hydrochloric acid and stored at −20°C for later analysis. Duplicates of 2 g of each fresh sample were placed into plastic test tubes, covered with parafilm, and stored at −20°C for later analysis of phenols and indoles. Fecal samples allocated for microbiota analysis were stored in 2 ml cryovials and stored at −80°C until analysis.

On 0 and 21 days of the experimental period, cats were fasted overnight, and a blood sample was collected to evaluate blood metabolites and health status. Cats were sedated before collecting 5 ml of blood *via* jugular venipuncture. For complete blood count analysis, 1 ml of blood from each cat was placed in EDTA vacutainer tubes and 4 ml was placed in serum separator tubes (Becton, Dickinson and Company, Franklin Lakes, NJ). Blood analyses were completed by the Clinical Pathology Laboratory at the University of Illinois College of Veterinary Medicine (Urbana, IL).

Experimental diets were subsampled and ground in a Wiley mill (model 4; Thomas Scientific, Swedesboro, NJ) using a size 10-mesh screen resulting in 2 mm average particle size used for proximate laboratory analysis. Total fecal samples were composited for each animal and partially dried in a forced air oven at 57°C. After drying, they were also ground in a Wiley mill to a 2-mm particle size.

### Chemical Analyses

After the diet and feces samples were prepared, DM and ash content were determined following the AOAC procedures [(6); methods 934.01 and 942.05). Crude protein concentration was evaluated using the Official Method of AOAC International by measuring total nitrogen with a LECO TruMac (model 630-300-300, Leco Corporation, St. Joseph, MI) (6). Fat content of the diet and fecal samples was determined using acid-hydrolysis and ether extraction following the methods of Budde and the American Association of Cereal Chemists (7, 8). Bomb calorimetry was utilized to determine the gross energy of the samples using a Parr 6200 calorimeter (Parr Instruments Co., Moline, IL). Further analysis of the fecal samples was completed to determine TDF content according to Prosky et al. and the Official Method of AOAC International (methods 985.29 and 991.43) (6, 9). Diet samples were analyzed using the same methods to determine TDF as well as soluble dietary fiber (SDF) and insoluble dietary fiber (IDF) contents.

Gas chromatography was used to measure SCFA and BCFA concentrations in the fresh fecal samples using a modified method of Sunvold et al. (10). These analyses were completed using a Hewlett-Packard gas chromatograph (Model 5890A Hewlett Packard, Avondale, PA) equipped with a flame ionization detector on a column (1.8 m × 4 mm i.d.) packed with GP 10% SP-1200/1% H_3_PO_4_ on 80/100 Chromosorb W AW (Supelco, Bellefonte, PA). Nitrogen was used as the carrier gas at a flow rate of 45 ml/min. Oven temperature was set at 125°C, the injection port at 175°C, and the detector port at 180°C. Fecal phenol and indole concentrations were measured using a Thermo Scientific TRACE 1300 Gas Chromatograph coupled with a FID in duplicate according to the modified procedure of Flickinger et al. (11). The internal standard used was 5-methylinodle. Following this method, 1-μl sample was injected at 220°C in splitless mode. A Nukol Supelco column (60 m length, 0.32 mm diameter) with a film thickness of 0.25 μm was used to separate the phenolic compounds. The oven temperature was held at 150°C for 1 min and then increased at 25°C per min until reaching 200°C and held at this temperature for 35 min. Ammonia concentration was measured according to the procedures of Chaney and Marbach (12).

### Microbial Analysis

Total DNA extraction from fresh fecal samples was completed using a Mo Bio PowerSoil kit (MO BIO Laboratories, Inc., Carlsbad, CA). A Qubit® 3.0 fluorometer (Life Technologies, Grand Island, NY) was used to quantify DNA concentration prior to amplification and sequencing. A Fluidigm Access Array (Fluidigm Corporation, South San Francisco, CA), in combination with Roche High Fidelity Fast Start Kit (Roche, Indianapolis, IN), was used for the amplification of the 16S rRNA gene. The primers 515F (5′-GTGYCAGCMGCCGCGGTAA-3′) and 806R (5′-GGACTACNVGGGTWTCTAAT-3′), targeting a 292-bp fragment of V4 region, were used for amplification (primers synthesized by IDT Corp., Coralville, IA) (13). A Fluidigm-specific primer, forward and reverse tags, was added in accordance with the Fluidigm protocol. A Fragment Analyzer (Advanced Analytics, Ames, IA) was used to verify the quality of amplicon region and size. A DNA pool was generated through a combination of equimolar amounts of the amplicons from each sample. The pooled samples were selected by size on a 2% agarose E-gel (Life Technologies, Grand Island, NY) and extracted using a Qiagen gel purification kit (Qiagen, Valencia, CA). The pooled, size-selected, and cleaned products were then run on an Agilent Bioanalyzer in order to confirm appropriate profile and mean size. The Roy J. Carver Biotechnology Center at the University of Illinois performed Illumina sequencing on a MiSeq using v3 reagent (Illumina Inc., San Diego, CA). A FASTX-Toolkit (version 0.0.14) removed the Fluidigm tags. Analysis of sequences was completed using QIIME 2.0 and DADA2 (version 1.14) (14, 15). The high-quality (quality value ≥ 20) sequence data, derived from the sequencing process, were demultiplexed. An open reference OTU clustered the sequences into operational taxonomic units (OTU), choosing against the SILVA 138 reference OTU database with a 97% similarity threshold (16). The OTUs observed fewer than two times (singletons), as well as OTUs with <0.01% of the total observations, were discarded. An average of 47,315 reads were obtained, with a total of 1,324,844 reads. The number of reads ranged from 39,416 to 56,474 per sample. To analyze for diversity and species richness, the dataset was rarified to 39,415 reads. Weighted and unweighted unique fraction metric (UniFrac) distances were performed by principal coordinate analysis (PCoA) (17).

### Statistical Analysis

Data were analyzed using the MIXED Model procedures of SAS version® 9.4 (SAS Institute Inc., Cary, NC). Animal was used as the random effect, and treatment diet was used as the fixed effect in the statistical model. Data normality was checked using the UNIVARIATE procedure, comparing all treatment least-square means. Experiment-wise error was controlled for using Tukey adjustment. The significance level was set at a probability of *P* < 0.05. Pooled standard errors of the mean also were obtained using the MIXED model procedure.

## Results

### Food Intake and Apparent Total Tract Macronutrient and Energy Digestibilities

The four treatment diets were formulated to contain similar nutrient composition (Table 1). This was confirmed through the chemical analysis of the diets (Table 2). Treatment did not have a significant effect on daily food intake (DM), wet fecal output (g/day), fecal DM output (g/day), fecal score, or fecal pH (*P* > 0.05) (Table 3). Additionally, the ATTD of DM, organic matter (OM), and crude protein (CP) were similar across treatments (*P* > 0.05). Dry matter digestibility ranged from 78.3 to 82.7%. Acid-hydrolyzed fat digestibility of CO (94.5%) was significantly higher compared with that of MF and MF + TP (91.7 and 91.2%, respectively) (*P* < 0.05) with BP being intermediate (92.6%). The BP diet had the highest TDF digestibility (54.2%) when compared with all the other treatments (average 22.1%) (*P* < 0.05). Digestible energy (kcal/g), which was calculated by subtracting fecal gross energy from diet gross energy, was higher for CO (3.94 kcal/g) than MF+TP (3.72 kcal/g) (*P* < 0.05), with MF and BP being intermediate.

**Table 2:** Chemical composition of treatments containing traditional and novel fiber sources for adult felines.

	**Treatment**^****1****^			
**Item**	**CO**	**MF**	**MF + TP**	**BP**
Dry matter, %	94.2	93.1	92.4	91.9
	% DM basis			
Organic matter	93.9	93.5	93.8	93.1
Ash	6.1	6.5	6.2	6.9
Acid hydrolyzed fat	17.6	16.7	16.2	16.2
Crude protein	31.3	30.9	29.6	30.3
Total dietary fiber	15.1	15.0	15.7	15.7
Soluble dietary fiber	3.4	3.8	3.2	6.1
Insoluble dietary fiber	11.7	11.2	12.5	9.6
Gross energy, kcal/g	4.7	4.6	4.6	4.5

1 *CO, cellulose; MF, M-fiber; MF + TP, M-fiber + tomato pomace; BP, beet pulp*.

**Table 3:** Food intake, fecal characteristics, and total tract apparent macronutrient digestibility of adult felines fed dietary treatments containing traditional and novel fiber sources.

**Item**	**Treatment**^****1****^				
	**CO**	**MF**	**MF + TP**	**BP**	**SEM^**2**^**
Food intake, as is	57.3	57.1	62.9	64.9	4.83
Dry matter, g/day	54.0	53.1	58.1	59.7	4.49
Fecal output, g/day (as is)	27.5	24.6	29.5	37.5	3.97
Fecal output, g/day (DM basis)	11.6	11.3	12.4	10.4	1.21
Fecal score	2.0	1.8	2.0	2.2	0.10
Fecal pH	7.7	7.5	7.3	7.1	0.21
**Digestibility, %**					
Dry matter	79.1	78.3	78.7	82.7	1.40
	% DM basis				
Organic matter	82.5	81.8	82.1	86.3	1.19
Acid hydrolyzed fat	94.5^a^	91.7^b^	91.2^b^	92.6^ab^	0.57
Crude protein	84.1	84.6	83.7	83.1	1.29
Total dietary fiber	21.8^b^	19.1^b^	25.5^b^	54.2^a^	5.44
Digestible energy, kcal/g	3.94^a^	3.74^ab^	3.72^b^	3.85^ab^	0.05

1 *CO, cellulose; MF, M-fiber; MF + TP, M-fiber + tomato pomace; BP, beet pulp.*

2 *Standard error of the mean.*

a, b *Means in the same row with different superscript letters are different (P < 0.05)*.

### Fecal Fermentative End Products

Cats fed the MF + TP and MF diets had significantly higher total fecal phenol and indole concentrations than CO- and BP-fed cats (*P* < 0.05) (Table 4). Fecal indole concentration followed the same pattern as total phenol and indole (*P* < 0.05), while fecal phenol concentration was not significantly affected (*P* > 0.05). Fecal ammonia concentration was also similar across all treatments (*P* > 0.05). BP resulted in the highest concentrations of total SCFA, acetate, and propionate (*P* < 0.05), while butyrate concentrations were similar for all treatments (*P* > 0.05). Total BCFA, isobutyrate, and isovalerate concentrations were higher in the MF + TP group than in the CO and BP groups (*P* < 0.05), with MF being intermediate. A similar trend for valerate concentration was observed with the MF + TP group being higher than the BP group (*P* < 0.05), with CO and MF groups being intermediate.

**Table 4:** Fecal fermentative end products for adult felines fed treatments containing traditional and novel fiber sources.

**Item (μmol/g DM basis)**	**Treatment**^****1****^				
	**CO**	**MF**	**MF + TP**	**BP**	**SEM^**2**^**
Total phenols/indoles	1.0^b^	1.8^a^	1.8^a^	0.6^b^	0.18
Phenols	0.0	0.1	0.1	0.1	0.01
Indoles	1.0^b^	1.7^a^	1.7^a^	0.5^b^	0.17
Total short-chain fatty acids	168.0^b^	189.2^b^	256.5^b^	583.7^a^	42.87
Acetate	99.1^c^	121.3^bc^	161.4^b^	390.2^a^	27.13
Propionate	44.5^b^	42.4^b^	61.0^b^	161.5^a^	13.66
Butyrate	24.4	25.5	34.2	32.0	3.54
Total branched-chain fatty acids	13.1^b^	17.2^ab^	22.7^a^	12.1^b^	2.08
Isobutyrate	2.9^b^	3.6^ab^	4.6^a^	3.1^b^	0.35
Isovalerate	4.5^b^	5.3^ab^	7.4^a^	4.2^b^	0.65
Valerate	5.7^ab^	8.3^ab^	10.2^a^	4.8^b^	1.29
Ammonia, mg/g DM	1.2	1.4	1.7	1.4	0.15

1 *CO, cellulose; MF, M-fiber; MF + TP, M-fiber + tomato pomace; BP, beet pulp.*

2 *Standard error of the mean.*

*^a−c^Means in the same row with different superscript letters are different (P < 0.05)*.

### Fecal Microbiota

**Figure 1:**
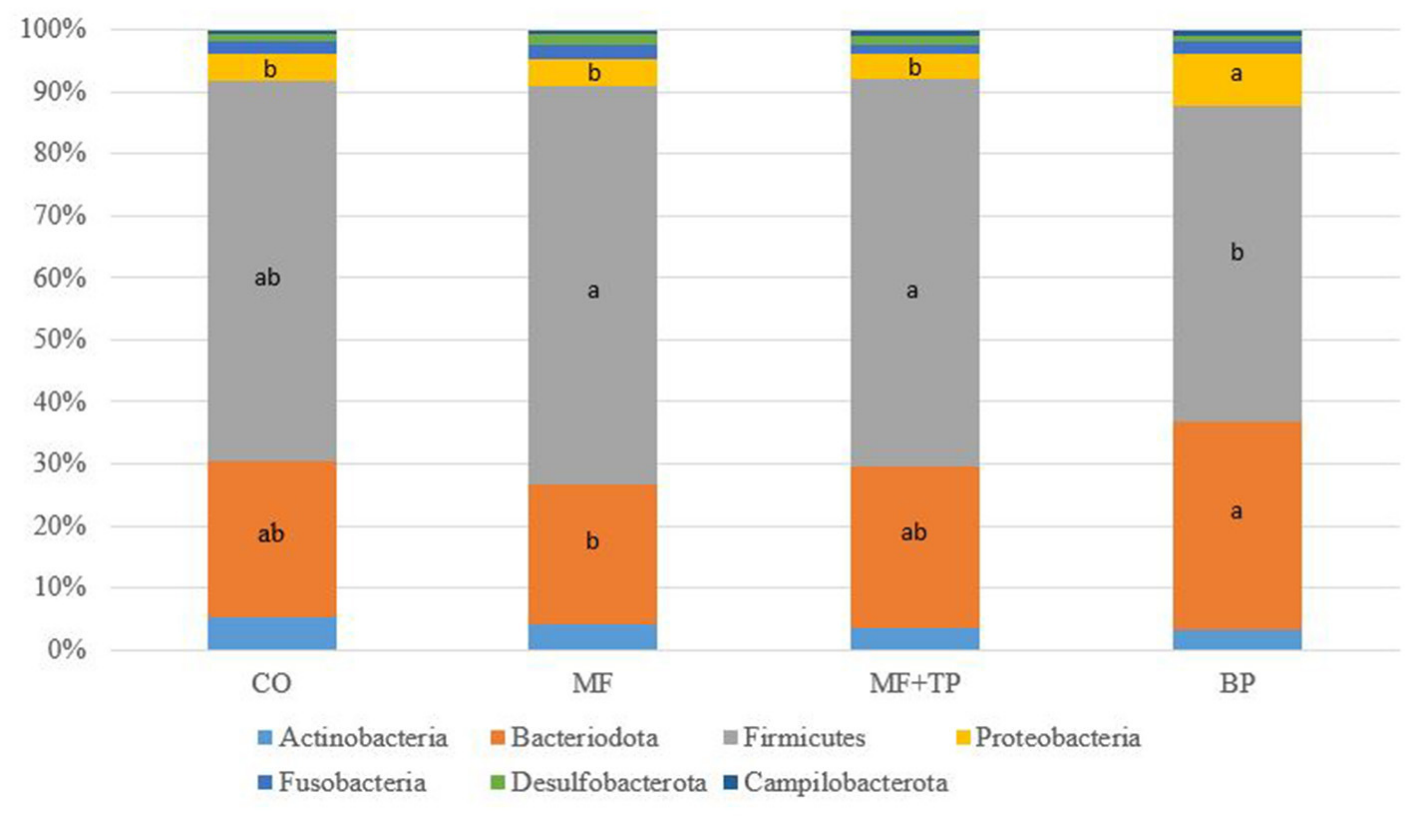
Fecal microbial composition at the phyla level for cats fed diets containing traditional and novel fiber sources.

The fecal microbiota composition for cats fed different dietary fibers was comprised of seven phyla (Figure 1) with Firmicutes, Bacteriodota, Proteobacteria, and Actinobacteria corresponding to more than 90% of the total sequences. Cats fed MF and MF + TP treatments had greater (*P* < 0.05) relative abundances of Firmicutes in relation to the BP treatment. Bacteriodota phylum was increased (*P* < 0.05) in cats fed BP in contrast with MF. Proteobacteria was increased (*P* < 0.05) in cats fed BP in contrast with all other dietary groups. A total of 38 families, 102 genera, and 66 species were identified; within those, over 10 and 20 taxonomic groups for family (Table 5) and genera (Table 6) differed (*P* < 0.05) among treatments, respectively. The relative abundance of Bacteroidaceae was greater (*P* < 0.05) for cats fed BP in comparison with those fed CO, but it did not differ for cats fed MF and MF + TP treatments. Cats fed BP also had greater (*P* < 0.05) relative abundance of Prevotellaceae in contrast with cats fed all the other dietary treatments. The relative abundance of Oscillospiraceae, Butyricicoccaceae, and Anaerovoracaceae were consistently higher (*P* < 0.05) in cats fed CO, MF, and MF + TP treatments in contrast with the BP treatment, whereas Succinivibrionaceae was consistently lower (*P* < 0.05) in cats fed those dietary treatments when compared with the BP treatment (Table 5).

**Table 5:** Fecal microbial composition (%) at the family level for cats fed treatments containing traditional and novel fiber sources.

**Phylum**	**Family**	**Treatment**^****1****^				
		**CO**	**MF**	**MF + TP**	**BP**	**SEM^**2**^**
Actinobacteria	*Bifidobacteriaceae*	0.75^a^	0.02^b^	0.26^ab^	0.53^ab^	0.178
	*Coriobacteriaceae*	3.97^a^	3.53^ab^	2.98^ab^	2.38^b^	0.431
Bacteroidota	*Bacteroidaceae*	9.40^b^	10.39^ab^	12.26^ab^	13.80^a^	1.280
	*Prevotellaceae*	12.33^b^	7.99^b^	9.50^b^	17.19^a^	1.536
	*Rikenellaceae*	0.40	0.62	0.60	0.02	0.207
	*Tannerellaceae*	1.54^ab^	1.88^ab^	2.10^a^	1.29^b^	0.232
Firmicutes	*Erysipelotrichaceae*	5.58^ab^	7.86^a^	7.14^ab^	4.97^b^	0.929
	RF39	0.03^b^	0.30^a^	0.00^b^	0.00^b^	0.084
	*Clostridiaceae*	1.13^ab^	0.41^b^	0.72^ab^	1.63^a^	0.355
	*Butyricicoccaceae*	1.53^a^	1.48^a^	1.38^a^	0.45^b^	0.241
	*Oscillospiraceae*	3.13^a^	3.21^a^	3.06^a^	0.93^b^	0.373
	*Eubacterium coprostanoligenes* group	0.68^a^	0.52^a^	0.51^a^	0.01^b^	0.171
	*Anaerovoracaceae*	2.13^a^	2.80^a^	2.44^a^	0.49^b^	0.469
Proteobacteria	*Succinivibrionaceae*	1.76^b^	0.84^c^	1.02^bc^	4.25^a^	0.282

1 *CO, cellulose; MF, M-fiber; MF + TP, M-fiber + tomato pomace; BP, beet pulp.*

2 *Standard error of the mean.*

*^a−c^Means in the same row with different superscript letters are different (P < 0.05)*.

**Table 6:** Fecal microbial composition (%) at the genus level for cats fed treatments containing traditional and novel fiber sources.

**Phylum**	**Genus**	**Treatment**^****1****^				
		**CO**	**MF**	**MF + TP**	**BP**	**SEM^**2**^**
Actinobacteria	*Bifidobacterium*	0.75^a^	0.02^b^	0.26^ab^	0.53^ab^	0.178
	*Collinsella*	3.97^a^	3.53^ab^	2.95^ab^	2.38^b^	0.431
Bacteroidota	*Bacteroides*	9.40^b^	10.39^ab^	12.26^ab^	13.80^a^	1.280
	*Paraprevotella*	0.01^b^	0.12^ab^	0.20^a^	0.00^b^	0.043
	*Prevotella*	9.22^b^	5.42^b^	6.03^b^	14.80^a^	1.460
	*Alistipes*	0.40^ab^	0.47^ab^	0.54^a^	0.02^b^	0.173
	*Parabacteroides*	1.54^ab^	1.88^ab^	2.10^a^	1.29^b^	0.232
	*Allobaculum*	0.46^ab^	0.75^a^	0.31^ab^	0.08^b^	0.193
Firmicutes	*RF39*	0.03^b^	0.30^a^	0.00^b^	0.00^b^	0.084
	*Clostridia UCG-014*	2.07^ab^	2.26^ab^	2.55^a^	1.42^b^	0.363
	*Butyricicoccus*	0.73^a^	0.56^ab^	0.45^ab^	0.19^b^	0.152
	*Butyricicoccaceae UCG-009*	0.74^a^	0.88^a^	0.82^a^	0.22^b^	0.177
	*Colidextribacter*	1.23^a^	1.16^a^	1.22^a^	0.58^b^	0.156
	*Oscillibacter*	0.61^a^	0.50^a^	0.44^a^	0.13^b^	0.103
	*Candidatus soleaferrea*	0.26^ab^	0.33^a^	0.30^a^	0.11^b^	0.065
	*Incertae sedis*	0.18^ab^	0.52^a^	0.29^a^	0.00^b^	0.087
	*Phocea*	0.22^a^	0.16^ab^	0.09^ab^	0.03^b^	0.056
	*Eubacterium coprostanoligenes* group	0.68^a^	0.52^a^	0.51^a^	0.01^b^	0.171
	*Mogibacterium*	0.24^ab^	0.29^ab^	0.43^a^	0.00^b^	0.138
	*Eubacterium brachy* group	0.39^a^	0.36^a^	0.22^ab^	0.08^b^	0.066
	*Eubacterium nodatum* group	0.78^ab^	0.90^a^	0.89^a^	0.32^b^	0.182
	*Megasphaera*	1.15^a^	0.77^ab^	1.26^a^	0.28^b^	0.290
Proteobacteria	*Succinivibrio*	1.76^b^	0.84^c^	1.02^bc^	4.25^a^	0.282

1 *CO, cellulose; MF, M-fiber; MF + TP, M-fiber + tomato pomace; BP, beet pulp.*

2 *Standard error of the mean.*

*^a−c^Means in the same row with different superscript letters are different (P < 0.05)*.

The relative abundance of Collinsella, a genus within the family Coriobacteriaceae and the phylum Actinobacteria, was greater (*P* < 0.05) in cats fed the CO treatment (4.0%) in contrast with BP (2.4%), and intermediate in cats fed the MF or MF + TP treatments (3.5 and 3.0%, respectively). In contrast, the relative abundance of Prevotella was greater in cats fed BP when compared with cats fed CO (9.2%), MF (5.4%), and MF + TP (6.0%). In addition, the relative abundance of several genera were consistently higher in cats fed CO, MF, and MF + TP, in contrast with BP including Clostridia UCG-014, Butyricicoccaceae UCG-009, Colidextrobacter, Oscillibacter, and Megasphaera. Cats fed BP (4.3%) had greater relative abundance of Succinivibrio than cats fed CO (1.8%) and MF + TP (1.0%), with MF (0.8%) being lowest (Table 6).

**Figure 2:**
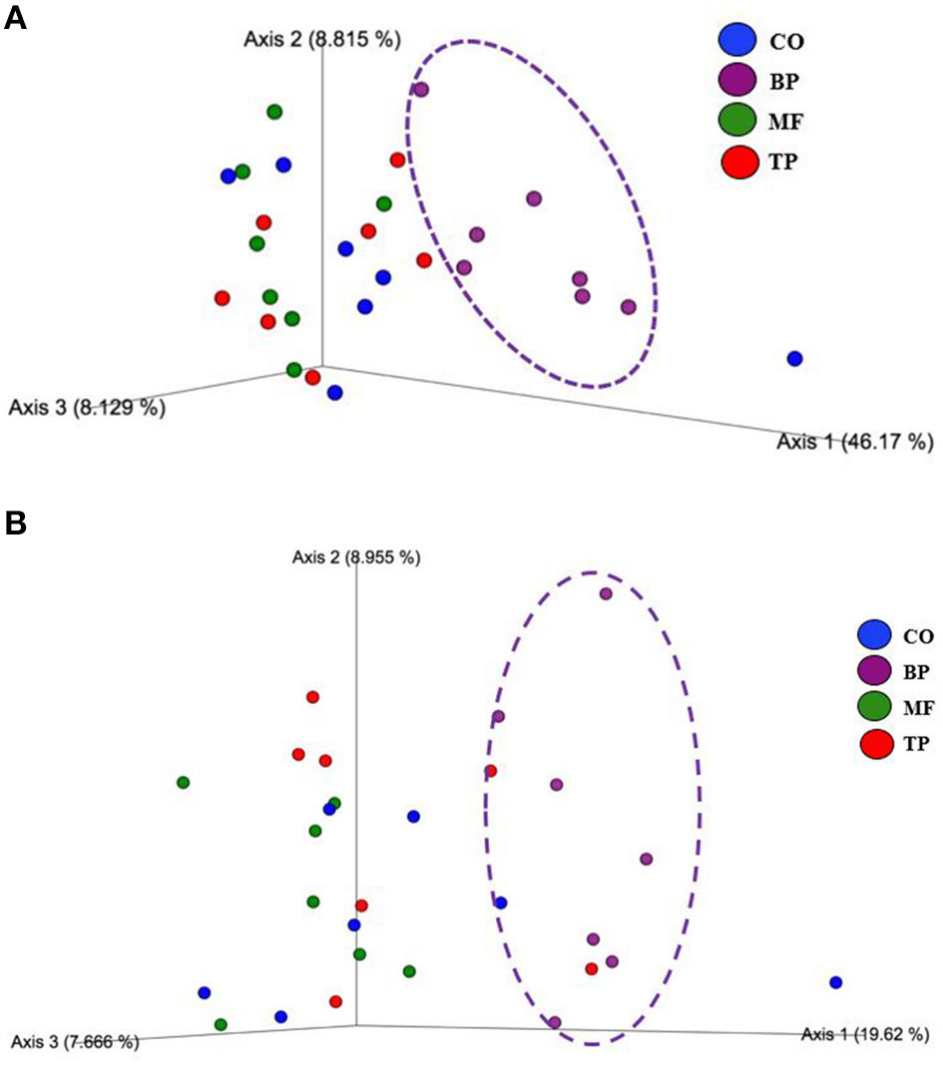
Principal coordinated plots of weighted **(A)** and unweighted **(B)** UniFrac distances of fecal microbial communities of cats fed diets containing traditional and novel fiber sources.

**Figure 3:**
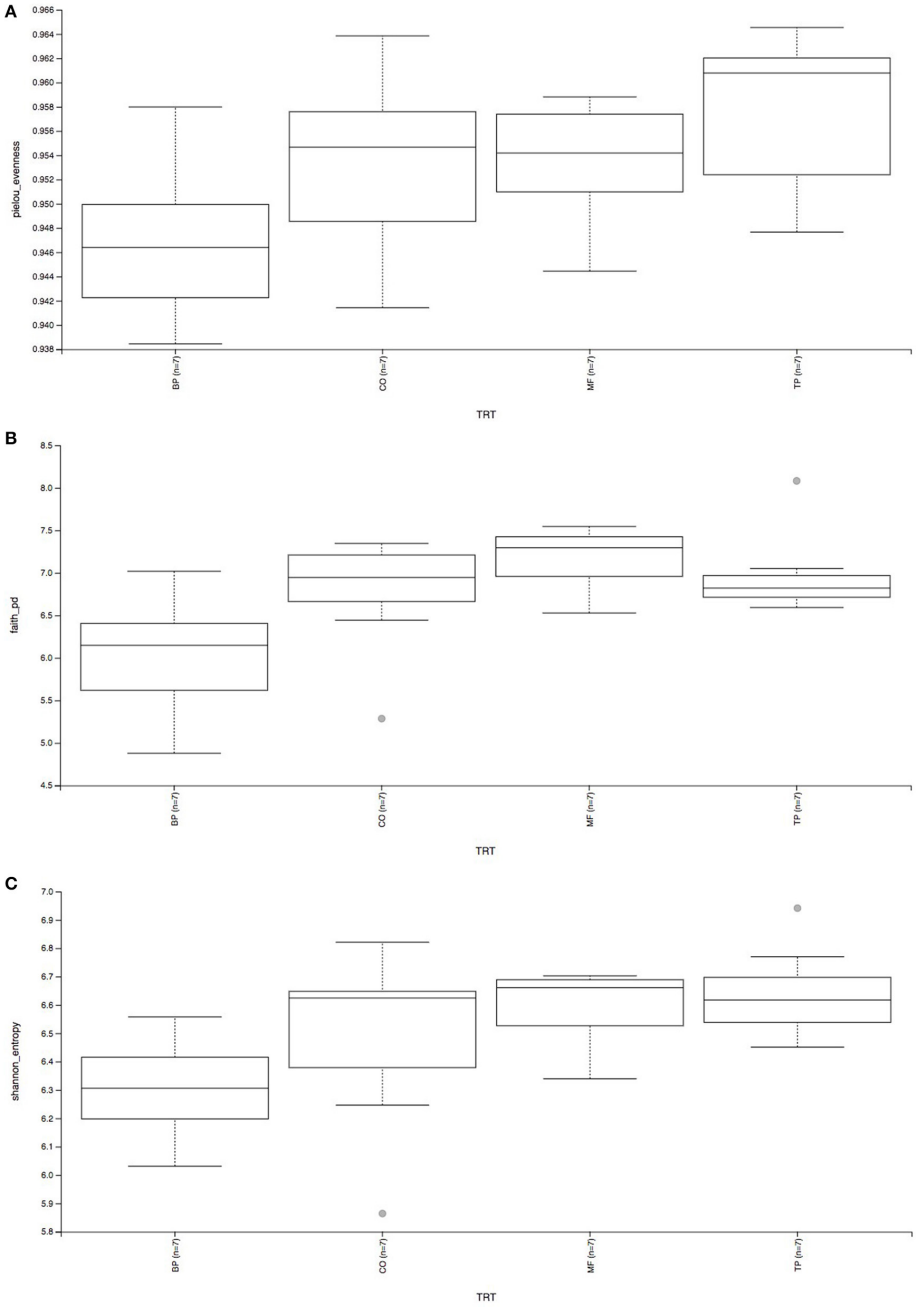
Alpha-diversity analysis of fecal microbial communities, measured by Pielou evenness **(A)**, Faith's phylogenetic diversity **(B)**, and Shannon entropy **(C)** of cats fed diets containing traditional and novel fiber sources.

Beta-diversity based on weighted (Figure 2A) and unweighted (Figure 2B) UniFrac analysis showed that fecal microbial community composition of cats fed the BP treatment differed (*P* and *q* value < 0.05) in comparison with cats fed CO, MF, and MF + TP treatments. Alpha-diversity was measured as Pielou evenness, Faith's phylogenetic diversity, and Shannon entropy (Figure 3). Fecal microbial diversity and richness based on the Pielou evenness index (Figure 3A) revealed that cats fed the BP treatment had lower α-diversity than cats fed the MF + TP treatment (*P* < 0.05 and *q* value < 0.1). Similarly, the α-diversity of cats fed BP was lower than cats fed other dietary treatments based on Faith's phylogenetic diversity (*P* and *q* value < 0.05; Figure 3B) and was also lower (*P* and *q* value < 0.05) than cats fed MF and MF + TP based on Shannon entropy index (Figure 3C).

### Serum Chemistry

Blood analysis was performed to determine the health status of the cats during the experimental period. Serum metabolites (Supplementary Material) were within normal ranges observed in healthy adult cats, and no treatment × time interaction or main treatment effect was observed. At baseline, however, the creatinine levels of cats assigned to the MF (1.7 mg/dl) and BP (1.6 mg/dl) treatments were slightly above the reference values. Similarly, cats fed CO (1.6 mg/dl), MF (1.8 mg/dl), and BP (1.6 mg/dl) diets, on 21 days of the experimental period, had slightly higher creatinine levels than the reference range (0.5–1.5 mg/dl).

## Discussion

### Diet, Food Intake, and Fecal Characteristics

The experimental diets all were formulated to maintain similar ingredients and nutrient composition, differing only in dietary fiber source (cellulose, beet pulp, *Miscanthus* grass fiber, and *Miscanthus* grass fiber and tomato pomace blend). Minimal variations in ingredient inclusion rates were necessary to obtain the targeted TDF content of 15%. This level of TDF was chosen to reflect the higher levels of commercial dietary fiber inclusion in order to emphasize any physiological responses to the various fiber ingredients. The chemical composition of the diets fell within a relatively narrow range, with the CO treatment having a slightly higher acid-hydrolyzed fat (AHF) and CP concentration, as well as a gross energy value. This could be due to the slightly higher inclusion level of poultry by-product meal in this diet. Overall, diets were very close to their target TDF content of 15%. Small variations are expected as TDF content can be affected by the plant's growing conditions, time of harvest, and plant maturity, among other things. The BP diet had higher levels of SDF and lower IDF as was expected based on the typical fiber profile of this ingredient (4). The CO, MF, and MF+TP diets were similar in SDF and IDF contents, which was also expected due to the similar fiber profile of cellulose and *Miscanthus* grass (18). It was predicted that the MF + TP blend might have slightly lower TDF, SDF, and IDF contents than MF as 2% tomato pomace was added at the expense of 2% of the *Miscanthus* grass fiber. The level of tomato pomace in the fiber blend was chosen to reflect the practical commercial inclusion of this ingredient in fiber matrices to increase SDF content. The composition of tomato pomace was reported by Swanson et al. as 56.9% TDF, 4.2% SDF, and 52.7% IDF (DM basis) in comparison with *Miscanthus* grass composition reported by Donadelli and Aldrich as 90% TDF, 7.3% SDF, and 82.7% IDF (DM basis) (18, 19). However, only lower SDF values were observed for the MF + TP blend compared with MF alone (3.2 and 3.8%, respectively), possibly due to the expected TDF variability of plant by-products that was previously mentioned or the low inclusion level of the tomato pomace.

Food intake (g/day) on an as-fed and DM bases did not differ among the treatment groups. Similarly, Donadelli and Aldrich saw no effect on food intake when cellulose, beet pulp, and *Miscanthus* grass were added to the formula at a 10% inclusion rate (20). Detweiler et al. evaluated diets including either 15.5% beet pulp (17.1% TDF), 9.6% cellulose (15.1% TDF), or 14% soybean hulls (16.6% TDF) and observed that cats fed the diet containing beet pulp had lower intake than the diet containing soybean hulls due to feed refusals (21). This indicates that although no effect on palatability was observed in this study with an inclusion level up to 9% *Miscanthus* grass (15.0% TDF), or up to 10% inclusion (13.8% TDF), higher inclusion levels and, subsequently, higher TDF contents may impact palatability and must be considered in the practical utilization of this ingredient (20).

Similar fecal scores were observed for all treatments ranging from 1.81 to 2.18 on a five-point scale. Previous research reported similar fecal scores with 8% inclusion of cellulose (11.2% TDF) and 12.5% inclusion of beet pulp (10.6% TDF), 1.8 and 2.3, respectively (10). Fecal output (g/day) on an as-is basis, as well as on a DM basis, were not significantly different among treatments. However, numerically, cats fed BP had the highest fecal output on an as-is basis and the lowest on a DM basis. This is due to the higher soluble fiber content of beet pulp that has a higher water-holding capacity, therefore increasing fecal water content and overall fecal mass. A similar effect was reported in felines by Detweiler et al. and in other studies across multiple species including canines and swine (21–23).

### Apparent Total Tract Macronutrient and Energy Digestibilities

Many studies have reported that dietary fiber sources can impact the digestibility of other macronutrients depending on their level of inclusion and fiber profile. However, DM and OM digestibility did not vary among dietary treatments in this study, and all diets were well-digested by adult cats. The DM and OM digestibility coefficients in the current research were reported to be just a few percentage units higher than similar treatments evaluated by Donadelli and Aldrich who compared diets with 10% inclusions of cellulose, beet pulp, and *Miscanthus* grass and observed that cats fed beet pulp (DM: 81.1%; OM: 85.9%) had significantly higher DM and OM coefficients than cellulose (DM: 75.5%; OM: 79.4%) and *Miscanthus* grass (DM: 76.2%; OM: 80.5%) (20). Kienzle et al. reported that the addition of dietary fiber significantly decreased OM digestibility by cats, and Sunvold et al. reported decreased OM and DM digestibility by cats when compared with a diet with no added fiber source (10, 24). The DM and OM digestibility coefficients reported by Sunvold et al. for the diet containing 12.5% beet pulp (DM: 80.4%; OM: 83.8%) and 8.1% cellulose (DM: 81.0%; OM: 83.5%) were similar to the values obtained for the diets in this study containing 11% beet pulp (DM: 82.7%; OM: 86.3%) and 7% cellulose (DM: 79.1%; OM: 82.5%) (10).

No difference in CP digestibility was detected among treatments in this study with a range of 83.1–84.6%. However, Donadelli and Aldrich observed the cellulose treatment to have a significantly higher CP digestibility than the beet pulp treatment with *Miscanthus* grass being intermediate (86.1, 85.8, and 84.2%, respectively) (20). In contrast, in this study, while not significant, the CP digestibility of the BP treatment was reported to be numerically lower than for all the other treatments. Many similar effects have been reported due to beet pulp's moderate level of fermentability compared with cellulose and other fiber sources with greater concentrations of insoluble fiber (10, 21, 25). Providing greater amounts of substrate for saccharolytic fermentation may result in increased microbial proliferation causing more microbial protein to be present in the feces. The quantification of this microbial nitrogen during analysis can lead to underestimations of actual crude protein digestibility.

The CO treatment resulted in higher AHF digestibility than the treatments containing *Miscanthus* grass fiber (MF and MF + TP). A similar effect was reported by Donadelli and Aldrich (20). The lipid content of the cellulose diet was slightly higher compared with the other treatments in both of these studies, which could have contributed to the higher digestibility. Another possible factor could be the higher lignin content of *Miscanthus* grass compared with cellulose or beet pulp, measured by Donadelli and Aldrich to be 13.68, 0.73, and 6.38%, respectively (18). Lignin has been reported to bind bile acids, inhibiting their action during lipid digestion, and potentially lowering fat digestibility (26). Digestible energy (kcal/g) followed the same pattern as AHF digestibility. The lower fat digestibility and DE can be beneficial tools in the development of diets for overweight and obese cats, which is a serious clinical condition in the pet population. According to these data, *Miscanthus* grass fiber may avoid further reductions in dietary fat content, which may assist maintaining palatability of weight management diets. This is important since weight management or loss diets tend to be formulated with higher concentrations of dietary fiber and lower fat content, resulting in poor acceptance, especially by cats. However, further studies should evaluate the impact of the utilization of *Miscanthus* grass fiber on fecal bile acid concentrations of cats. Since lignin can bind with bile acids in the gastrointestinal tract, it is possible that greater amounts of bile acids will be excreted in the feces, lowering their ability to recycle *via* enterohepatic circulation. This could lead to increased requirements of dietary taurine for cats, since taurine conjugates bile acids to form water-soluble bile salts.

Total dietary fiber ATTD was the highest for the cats fed BP than for all the other treatments. This was expected as beet pulp has been shown to be moderately fermented in the feline intestinal tract in comparison with cellulose, which has a low fermentative potential (10). Both treatments including *Miscanthus* grass fiber (MF: 19.1% and MF + TP: 25.5%) were similar to cellulose (21.8%) in this regard, as they had greater IDF content, which is poorly fermented and, therefore, excreted in higher quantities in the feces. Donadelli and Aldrich reported a similar TDF digestibility coefficient (20.8%) with the inclusion of 10% *Miscanthus* grass (20). While not significantly different, the MF + TP treatment had a numerically higher TDF digestibility than did MF and CO treatments. This could be due to the inclusion of tomato pomace in the fiber blend that was reported by Swanson et al. to have a higher fermentation potential than cellulose using an *in vitro* model with canine fecal inoculum (19).

### Fecal Fermentative End Products and Microbiota

Short-chain fatty acids are the major organic end products of saccharolytic fermentation, with increased concentration indicating increased fermentative processes. While not an entirely accurate representation of complete SCFA production in the large intestine, fecal SCFA concentration has been utilized by researchers as a non-invasive method of estimating the production of these fermentative end products by the gut microbiota (27). Total fecal SCFA concentration was the highest in the BP group (583.7 μmol/g, DM basis) compared with all the other treatments. Detweiler et al. evaluated higher levels of beet pulp (15.5% inclusion; 17.1% TDF) that resulted in higher levels of total SCFA (699.7 μmol/g, DM basis), and also observed the beet pulp treatment to produce the highest total SCFA compared with cellulose and soybean hulls (21). Fischer et al. reported similar results when evaluating a diet including 15.5% beet pulp (26% TDF) in overweight cats (28). This increased production of SCFA indicates that beet pulp has higher fermentability compared with the other fiber substrates evaluated, which is supported by the findings of Sunvold et al. who observed that beet pulp had a higher OM disappearance and total SCFA production than did cellulose in an *in vitro* assay using feline fecal inoculum (10). A decrease in gut lumen and fecal pH also is associated with higher fermentative activity as the buildup of these metabolites starts to acidify the environment. However, no difference in fecal pH was observed, with values ranging from 7.1 (BP) to 7.7 (CO).

When evaluating the fecal SCFA on an individual basis, the same trend was observed for fecal acetate and propionate concentrations, being highest for the BP group. Our findings also are supported by Detweiler et al. who reported that cats fed beet pulp had significantly higher fecal concentrations of acetate (459.2 μmol/g, DM basis) and propionate (139.0 μmol/g) compared with cats fed diets with no additional fiber, cellulose, or soybean hulls (average acetate 219.6 μmol/g; average propionate 62.0 μmol/g) (*P* < 0.05) (21). Fischer et al. also observed that when compared with diets containing wheat bran and sugarcane fiber and a diet with no added fiber source (average acetate 217 mM/kg DM; average propionate 95.7 mM/kg), cats fed beet pulp had significantly higher fecal concentrations of acetate (427 mM/kg) and propionate (214 mM/kg) (*P* < 0.05) (28). No statistical differences were observed in fecal butyrate concentration across treatments. However, CO and MF treatments had numerical values (24.4 and 25.5 μmol/g, respectively) that grouped closer together, while MF + TP and BP treatments also were more closely grouped (34.2 and 32.0 μmol/g). It is well-established that SCFAs play a significant role in maintaining gastrointestinal health as they provide energy to colonocytes, reduce inflammation, and have been implicated in the inhibition of cancer (29). While the available substrate is an important factor affecting SCFA production, complex factors such as the removal of fermentative wastes and microbial population composition also play a critical role and are important to consider when evaluating the relationships between dietary components and fermentative metabolites (29).

The fermentation of protein by microbiota in the large intestine results in end products such as ammonia, phenols, indoles, and BCFA. Increases in these compounds often are seen as a negative outcome as they are considered putrefactive compounds that can lead to unwanted fecal malodor (30). No difference was observed in fecal ammonia (1.4–1.7 mg/g DM) or phenol concentration (0.037–0.053 μmol/g, DM) among treatments. Detweiler et al. also reported no significant difference in these concentrations among treatments including beet pulp, cellulose, and soybean hulls as fiber sources and a treatment with no added fiber source (21). Total phenol and indole and individual indole concentrations followed similar patterns of being higher in the treatment groups containing *Miscanthus* grass fiber (MF and MF + TP). While the indole compound can help to maintain gut homeostasis by promoting barrier functions, regulating inflammation, and possibly aid in satiety, it can also be metabolized into indoxyl sulfate, which is a uremic toxin that has been associated with negative health outcomes in humans such as cardiovascular disease and chronic kidney disease (29). Barry et al. reported higher indole concentrations (1.4 μmol/g, DM) with lower inclusion levels of cellulose (4% inclusion; 7.9% TDF), while Detweiler et al. reported lower indole concentrations (0.7 μmol/g, DM) with higher inclusion levels of cellulose (9.6%; 15.1% TDF) (21, 31). The inclusion level of cellulose and the indole concentration in the current study (7% inclusion; 0.97 μmol/g, DM) were intermediate to the values reported in previous studies. In contrast, Detweiler et al. observed a higher level of indole (1.4 μmol/g, DM) with a higher inclusion rate of beet pulp (15.5% inclusion; 17.1% TDF) (21). Overall, the range of indole concentration observed across treatments in this study was similar to the ranges reported in other studies evaluating healthy adult cats (21, 31).

Increased BCFA concentration indicates that higher levels of peptides and amino acids are present in the large intestine and are available for fermentation. Cats fed the MF + TP diet had greater total BCFA concentrations than cats fed CO and BP treatments. Previous research by Barry et al. indicated that the addition of rapidly fermentable fibers (fructooligosaccharides and pectin) increased total BCFA concentrations compared with cellulose (31). This could explain the effect reported with the addition of tomato pomace in the MF + TP fiber blend, as higher levels of rapidly fermented pectin are generally observed in fruit by-products (4). In contrast, the total BCFA concentrations reported by Barry et al. were much higher (44.0–63.9 μmol/g, DM) with low inclusions (4%) of cellulose, fructooligosaccharides (FOS), and pectin than those observed in the current study (12.06–22.68 μmol/g, DM) (31).

The use of dietary fiber has been an effective strategy in the modulation of gut microbiota to support gastrointestinal and systemic host health. Metabolites produced by gut microbiota (e.g., SCFA) are involved in the beneficial health effects on the host. These metabolites have been described as post-biotics, a term that is ill-defined by the scientific community (32, 33). The domestic cat, despite being a strict carnivore and having a short and unsacculated colon, has considerable capacity for hindgut microbial fermentation and production of fermentative end products (34). In companion animal nutrition, a few studies have evaluated the effects of dietary fiber in the modulation of gut microbiota in cats (35–41); however, most of those studies evaluated the effects of soluble and (or) prebiotic sources [e.g., FOS, lactosucrose, pectin, xylooligosaccharides (XOS)] in contrast to cellulose or a no-added fiber diet. Thus, the effect of *Miscanthus* grass fiber and tomato pomace on fecal microbiota has not been evaluated previously. Characterization of the feline gut microbiota has shown that Firmicutes, Bacteroidetes, Proteobacteria, and Actinobacteria are dominant phyla in adult healthy cats (38, 39, 42, 43). Our findings agree with current literature, even though the relative abundance of each phylum may differ among individuals and based on experimental methods used.

The phyla Firmicutes, Bacteroidetes (Bacteroidota), and Actinobacteria are considered important producers of metabolites that have direct beneficial effect on gut and host health (44). A recent study evaluating the effects of dietary XOS supplementation on the fecal microbiota of healthy cats reported that diets containing either 0.04 or 0.4% of XOS, at the expense of cellulose, resulted in increased relative abundance of Collinsella (2.6–4.4%) and decreased abundance of Megasphaera (0.80–0.82%) in contrast with cats fed a control diet containing 1% cellulose (1.7 and 1.3%, respectively) (41). A similar relative abundance of Megasphaera was observed in cats fed CO, MF, and MF + TP diets (range: 0.8–1.3%) in this study. Lyu et al. also reported increased relative abundance of Ruminococcaceae, Erysipelotrichaceae, and Lachnospiraceae in contrast to cats fed the control diet (41). In the current study, cats fed CO, MF, and MF + TP treatments had increased relative abundance of Allobaculum, a genus within the Erysipleotrichaceae family, and a few genera within the family Ruminococcaceae (i.e., Candidatus Soleaferrea, Incertae Sedis, and Phocea). Garcia-Mazcorro et al. reported increased relative abundance of Veilonellaceae and decreased relative abundance of Gammaproteobacteria in fecal samples of healthy cats during FOS and inulin supplementation (40). In the current study, a greater relative abundance of Megasphaera, a genus within the family Veilonellaceae, and a lower relative abundance of Succinivibrio, a genus within the family Succinivibrionaceae and class Gammaproteobacteria, were observed in fecal samples of cats fed CO, MF, or MF + TP treatments in contrast with cats fed the BP treatment. More recently, Butowski et al. evaluated the fecal microbial communities of cats fed kibble, raw, and raw + fiber diets (45). The fiber sources included in the kibble diet were beet pulp and inulin (% inclusion not provided), and in the raw + fiber diet 2% of inulin and 2% of cellulose were included (as-is basis). In general, lower relative abundance of Collinsella (0.03%) and Bacteroides (0.2%) and greater relative abundance of Prevotella were reported for cats fed the kibble diet in comparison with our findings. The relative abundance of Succinivibrio of cats fed BP (4.3%) was greater than the relative abundance of cats fed the kibble diet (1.2%) as reported by Butowski et al. (45). Differences in the relative abundance of microbial taxa among studies can be affected by many variables including animal variation and differences in methods including DNA extraction, variable region and primers used for sequencing, bioinformatic procedures, and the reference database utilized.

From this study, it is clear that different dietary fibers exert distinctive effects on the modulation of the feline gut microbiota. Cats fed CO, MF, and MF + TP treatments had greater microbial taxa similarities among them, in contrast with cats fed the BP treatment. This effect was evident based on the presence and absence of particular taxa (unweighted UniFrac), as well as their relative abundance (weighted UniFrac). Microbial diversity has been used as an indicator of gut health, as lower microbial diversity has been associated with clinical conditions including irritable bowel disease and small cell lymphoma in cats (46). Therefore, increased α-diversity observed in cats fed diets containing *Miscanthus* grass fiber may support gut health by maintaining microbial richness and evenness in adult cats. However, healthy cats supplemented with either FOS or apple pomace had decreased α-diversity when compared with baseline values (47). Overall, there were apparent microbial benefits across all dietary treatments. In addition, all cats in this study were healthy, and therefore, microbial shifts should be evaluated on different dietary fiber sources and amounts may be used to modulate gut microbiota and their metabolites in the hindgut of felines.

### Serum Chemistry

Serum chemistry and complete blood count analysis were within reference ranges for healthy adult cats. Creatinine was an exception and observed to be slightly higher than the reference range for MF and BP treatments at baseline and for CO, MF, and BP treatments at the end of the trial period. These deviations from the reference range were small and could be due to individual variation among cats. No effect of treatment was observed. Additionally, glucose concentrations were above the normal range. However, this has been observed as a side effect of the sedation used during the blood collection. The results of the serum chemistry and complete blood cell count (data not provided) and the lack of clinical symptoms indicate that the treatments did not result in any negative health outcomes.

### Implications

The findings from this research indicate that *Miscanthus* grass fiber is an advisable dietary fiber for adult felines. The addition of *Miscanthus* grass fiber and the MF + TP blend had no detrimental effects on animal health, fecal quality, or macronutrient digestibility. Diet inclusion of *Miscanthus* grass fiber up to 9% (15% TDF) had no negative effect on voluntary food intake, indicating that it had acceptable palatability to the cats. The resulting concentrations of fecal fermentative end products were more similar to those observed in the CO group than in the BP group, as expected from the similarities in the fiber profile of these ingredients. In conclusion, *Miscanthus* grass can be utilized by the pet food industry as an economical and environmentally conscious ingredient that can provide flexibility in the formulation of diets that aim to maximize the health benefits of dietary fiber. *Miscanthus* grass fiber can be effectively used as a base ingredient to develop fiber blends in combination with more soluble and fermentable dietary fiber, including prebiotic sources, which might be beneficial to improve SCFA production and modulate gut microbiota. In this study, inclusion of 2% TP in combination with MF resulted in small numerical increases in fecal SCFA concentrations, suggesting that fiber blends can be used to support gut and host health. Inclusion of dietary fibers was an effective strategy to modulate feline gut microbiota. Cats fed diets containing *Miscanthus* grass fiber had greater α-diversity than cats fed BP. Future studies should further evaluate nutraceutical uses, additional fiber blends, and diet formats, as *Miscanthus* grass fiber can be a functional ingredient in multiple dietary platforms, including weight management, gut health, and hairball control.

## Data Availability Statement

The data generated for the study are deposited in the Illinois Data Bank repository, accession number (doi: 10.13012/B2IDB-3595148_V1).

## Ethics Statement

The animal study was reviewed and approved by University of Illinois Animal Care and Use Committee.

## Author Contributions

MdG designed the experiment. SF and FH performed the laboratory analyses. SF and MdG performed the statistical analyses and wrote the manuscript. BS and SR-Z performed the bioinformatics analysis for fecal microbial analysis. All authors revised and provided intellectual input on this manuscript.

## Conflict of Interest

The authors declare that the research was conducted in the absence of any commercial or financial relationships that could be construed as a potential conflict of interest.
