# Supplementary material for: Miscanthus Grass as a Novel Functional Fiber Source in Extruded Feline Diets
Source: Front Vet Sci. 2021 Jun 4;8:668288. doi: 10.3389/fvets.2021.668288 (PMC8213067; doi:10.3389/fvets.2021.668288)
Supplement: Supplementary file 1 [file Table_1.docx]

Supplementary Material

# Supplementary Table 1. Serum metabolites of adult felines fed treatments containing traditional and novel fiber sources

|  |  | ***Day 0*** | | | |  | ***P* – Value** | | |
| --- | --- | --- | --- | --- | --- | --- | --- | --- | --- |
| Item | Reference Range^2^ | CO^1^ | MF | MF+TP | BP | SEM^3^ | Trt | Day | Trt*Day |
| Creatinine (mg/dL) | 0.5-1.5 | 1.5 | 1.7 | 1.4 | 1.6 | 0.08 | 0.07 | 0.03 | 0.82 |
| BUN^3^ (mg/dL) | 6.0-30.0 | 21.9 | 21.9 | 20.3 | 21.1 | 0.79 | 0.30 | 0.72 | 0.72 |
| Total protein (g/dL) | 5.1-7.0 | 6.4 | 6.3 | 6.5 | 6.3 | 0.12 | 0.83 | 0.20 | 2.30 |
| Albumin (g/dL) | 2.5-3.8 | 3.3 | 3.4 | 3.2 | 3.3 | 0.06 | 0.37 | 0.01 | 0.02 |
| Globulin (g/dL) | 2.7-4.4 | 3.1 | 2.9 | 3.3 | 3.0 | 0.11 | 0.14 | 0.32 | 0.27 |
| Ca (mg/dL) | 7.6-11.4 | 9.1 | 9.0 | 9.1 | 9.0 | 0.10 | 0.83 | <.01 | 0.22 |
| P (mg/dL) | 2.7-5.2 | 4.8 | 4.9 | 5.1 | 4.9 | 0.21 | 0.64 | 0.64 | 0.98 |
| Na (mmol/L) | 141-152 | 147.1 | 147.0 | 146.6 | 145.9 | 0.60 | 0.79 | <.01 | 0.07 |
| K (mmol/L) | 3.9-5.5 | 4.1 | 4.1 | 4.2 | 4.2 | 0.18 | 0.45 | <.01 | 0.11 |
| ALT (SGPT) | 8.0-65.0 | 49.7 | 41.4 | 46.3 | 51.7 | 6.67 | 0.69 | 0.01 | 0.78 |
| Cl (mmol/L) | 107-118 | 118.3 | 118.3 | 117.7 | 117.7 | 0.62 | 0.66 | <0.01 | 0.13 |
| Glucose (mg/dL) | 68-126 | 146.6 | 152.9 | 151.1 | 167.0 | 14.85 | 0.54 | 0.21 | 0.10 |
| Total Bilirubin (mg/dL) | 0.1-0.3 | 0.1 | 0.1 | 0.1 | 0.1 | 0.01 | 0.25 | 0.67 | 0.57 |
| Total Cholesterol (mg/dL) | 66-160 | 140.6 | 158.0 | 146.9 | 157.0 | 13.09 | 0.91 | 0.14 | 0.10 |
| Triglycerides (mg/dL) | 32-154 | 34.6 | 40.6 | 44.7 | 39.0 | 4.45 | 0.54 | 0.05 | 0.67 |
| Bicarbonate (mmol/L) | 16-24 | 19.1 | 18.7 | 19.3 | 18.6 | 0.47 | 0.16 | <0.01 | 0.23 |

**Supplementary Table 1 (cont.)** Serum metabolites of adult felines fed treatments containing traditional and novel fiber sources

|  |  | ***Day 21*** | | | |  | ***P* - Value** | | |
| --- | --- | --- | --- | --- | --- | --- | --- | --- | --- |
| Item | Reference Range^2^ | CO^1^ | MF | MF+TP | BP | SEM^3^ | Trt | Day | Trt*Day |
| Creatinine (mg/dL) | 0.5-1.5 | 1.6 | 1.8 | 1.5 | 1.6 | 0.08 | 0.07 | 0.03 | 0.82 |
| BUN^3^ (mg/dL) | 6.0-30.0 | 22.1 | 21.7 | 20.0 | 21.7 | 0.79 | 0.30 | 0.72 | 0.72 |
| Total protein (g/dL) | 5.1-7.0 | 6.3 | 6.3 | 6.5 | 6.5 | 0.12 | 0.83 | 0.20 | 2.30 |
| Albumin (g/dL) | 2.5-3.8 | 3.2 | 3.4 | 3.3 | 3.4 | 0.06 | 0.37 | 0.01 | 0.02 |
| Globulin (g/dL) | 2.7-4.4 | 3.0 | 2.9 | 3.2 | 3.1 | 0.11 | 0.14 | 0.32 | 0.27 |
| Ca (mg/dL) | 7.6-11.4 | 9.3 | 9.4 | 9.4 | 9.2 | 0.10 | 0.83 | <.01 | 0.22 |
| P (mg/dL) | 2.7-5.2 | 4.7 | 4.9 | 5.1 | 5.0 | 0.21 | 0.64 | 0.64 | 0.98 |
| Na (mmol/L) | 141-152 | 148.0 | 149.0 | 148.0 | 149.3 | 0.60 | 0.79 | <.01 | 0.07 |
| K (mmol/L) | 3.9-5.5 | 4.0 | 3.5 | 4.0 | 3.7 | 0.18 | 0.45 | <.01 | 0.11 |
| ALT (SGPT) | 8.0-65.0 | 61.1 | 54.4 | 51.0 | 58.9 | 6.67 | 0.69 | 0.01 | 0.78 |
| Cl (mmol/L) | 107-118 | 116.7 | 116.7 | 116.1 | 118.0 | 0.62 | 0.66 | <0.01 | 0.13 |
| Glucose (mg/dL) | 68-126 | 175.3 | 139.4 | 130.3 | 118.7 | 14.85 | 0.54 | 0.21 | 0.10 |
| Total Bilirubin (mg/dL) | 0.1-0.3 | 0.1 | 0.1 | 0.1 | 0.1 | 0.01 | 0.25 | 0.67 | 0.57 |
| Total Cholesterol (mg/dL) | 66-160 | 159.9 | 156.1 | 147.3 | 158.7 | 13.09 | 0.91 | 0.14 | 0.10 |
| Triglycerides (mg/dL) | 32-154 | 31.7 | 39.3 | 37.6 | 35.9 | 4.45 | 0.54 | 0.05 | 0.67 |
| Bicarbonate (mmol/L) | 16-24 | 18.3 | 18.6 | 18.6 | 16.9 | 0.47 | 0.16 | <0.01 | 0.23 |

^1^ CO = Cellulose; MF = M-Fiber; MF+TP = M-Fiber + Tomato Pomace; BP = Beet Pulp

^2^ References ranges were provided by the University of Illinois Veterinary Diagnostic Laboratory

^3^ SEM = Standard error of the mean; BUN = Blood urea nitrogen
